# Duoethnography as a dialogic and collaborative form of curriculum inquiry for resident professionalism and self-care education

**Published:** 2018-07-27

**Authors:** Susan Docherty-Skippen, Karen Beattie

**Affiliations:** 1 Faculty of Education, Brock University, Ontario, Canada; 2 Department of Medicine, McMaster University, Ontario, Canada

## Abstract

Medical residency is an important time in the development of physician professionalism, as residents’ identities and medical responsibilities shift from student-learners to practitioner-leaders. During this transition time, many residents struggle with stress due to the unique pressures of their post-graduate training. This, in turn, can potentially hinder successful professional identity development. In response, the Royal College of Physicians and Surgeons of Canada (RCPSC) has incorporated physician health into its CanMEDS professional competency framework. Although this framework identifies enabling self-care professional competencies (e.g., capacity for self-regulation and resilience for sustainable practice), it does not specify the types of educational strategies best suited to teach and assess these competencies. To support the prevention and rehabilitation of resident health issues, residency training programs are faced with the complex challenge of developing socially accountable curricula that successfully foster self-care competencies. Duoethnography, a dialogic and collaborative form of curriculum inquiry, is presented as a pedagogical model for resident professionalism and self-care education. Merits of duoethnography centers on its: 1) capability to foster self-reflexive and transformative learning; 2) versatility to accommodate learner diversity; and 3) adaptability for use in different social, situational, and ethical contexts.

## Introduction

Medical residency is an important time in the development of physician professionalism, as residents’ identities and medical responsibilities shift from student-learners to practitioner-leaders. For this reason, duoethnography, a dialogic and collaborative form of curriculum inquiry, is presented as a pedagogical model for resident professionalism and self-care education. Merits of duoethnography centers on its: 1) capability to foster self-reflexive and transformative learning; 2) versatility to accommodate learner diversity; and 3) adaptability for use in different social, situational, and ethical contexts.

### Residency as a transitory time during professional identity development

“Identity only becomes an issue when it is in crisis, when something assumed to be fixed, coherent and stable is displaced by the experience of doubt and uncertainty.”^1^

Although it is a dynamic and iterative process, a physician’s professional identity typically develops during the time they enter medical school and transitions into residency, until the time they move into professional practice.^2-3^ While it is acknowledged that residency is a transitory time during which residents focus more heavily on the development of medical expertise at the cost of personal life balance, the unique challenges of resident post-graduate training have been found to trigger distress.^4-7^ Stressors include worries about high financial debt load, extended work hours, sleep deprivation, licensing exams, complex and challenging patient caseloads, repeated exposure to grief and loss, and harassment from staff physicians, allied health workers, patients, and even other residents in training.^4-15^ Compounding these issues are the real and/or perceived fears related to social stigma, negative training evaluations, and jeopardized career opportunities that prevent residents from accessing psychological support/counselling services to help with the management of their personal and professional stress.^4,16^ In some cases, this added stress burden leads to burnout.^4,6,9^ Resident burnout has been defined as emotional exhaustion, depersonalization, and reduced accomplishment.^9^ Often, it manifests in poor academic performance and substandard patient care.^11-15,17-18^ When this happens, residents are more likely to experience crises in professional identity development, situations where their actions do not match who they are or who they want to become.^19^ These identity crises can reinforce feelings of fear, failure, lack of self-confidence, and lead to professional self-doubt.^19^ Since physicians’ professional identities are largely shaped by the social and cultural expectations placed upon them, the way residents are taught to examine these critical moments and sources of stress are intrinsically connected to the way they view themselves in a professional and self-caring capacity.^19^

### Self-care and the CanMEDS professional competency framework

In response to the concerns about physician/resident health and wellness, the Royal College of Physicians and Surgeons of Canada (RCPSC) has included self-care as an essential component of its CanMEDS curriculum competency framework.^20^ The CanMEDS framework forms the basis for all RCPSC education accreditation standards and specialty training programs including postgraduate residency. It provides “a comprehensive definition of the abilities needed for all domains of medical practice.”^21^ These abilities are systematized into seven categories of competencies expressed as physician roles; one of these roles is professionalism. According to CanMEDS; “as professionals, physicians are committed to the health and well-being of individual patients and society through ethical practice, high personal standards of behaviour, accountability to the profession and society, physician-led regulation, and maintenance of personal health.”^21^ Explicit best-practice guidance for the instruction and assessment of resident professionalism and self-care competencies is lacking in the CanMEDS framework and in the medical professionalism literature in general.^21^

Medical educators have reported substantial challenges in developing and implementing effective educational strategies related to professionalism and self-care competency in medical/resident curriculum.^22-26^ In part, some of these challenges relate to the multifaceted social dynamics of professional identity development and personal well-being, and other challenges relate to the suitability of teaching methods and evaluation criteria reflective of its tacit complexity.^2,3,24-26^ Typical teaching and assessment strategies used to instill and assess cognitive and psychomotor knowledge in medical education/resident training (i.e., standardized practicums, high-fidelity simulations, knowledge tests, and in-training evaluations), do not accurately gauge learning effectiveness in the affective knowledge domain (i.e., knowledge of self, development of attitudes, values, self-care, and self-efficacy).^22-27^ Compounding this issue, there is a paucity of research that discusses strategies for successful professional identity development and self-care competency in medical education/residency training. A large portion of the research on resident/physician health and well-being has centered on its negative outcomes, (i.e., stress and burnout), rather than on positive ways to support the prevention and rehabilitation of resident/physician health issues in relation to the RCPSC’s educational standards of professional competency.^4-15^ Fostering residents to become “wholly engaged physicians,” who are cognisant of and connected to the care needs of both their patients and themselves, is important for physician sustainability.^14^

### Duoethnography as a dialogic and collaborative form of curriculum inquiry

Although first developed as a qualitative research method to study how individuals understand common experiences, duoethnography is a dialogic and collaborative form of curriculum inquiry.^28-30^ It has proven to be a successful pedagogical tool in the academic support and professional identity development of graduate students experiencing stress.^31^ Similar to reflective practice, duoethnography situates knowledge in personal perspective, then fosters the development and transformation of that knowledge over time.^28^ Where duoethnography differs from other forms of reflection that focus on *introspection* (i.e., an examination of one’s own conscious beliefs and emotions), duoethnography invites *extrospection* (i.e., an examination of self through a negotiated consideration and thoughtful observation, with others, of things external to one’s self). This allows learners from diverse cultural and situational backgrounds to understand and appreciate themselves differently within the larger social, cultural, and political contexts in which they are immersed.

Although guided by eight principles, (refer to table 1), duoethnography does not prescribe to a set formula of design.^28-30^ Instead, “each group of researchers [participants] can and will adapt the method to their unique circumstances using its basic principles as a guide.”^28^ This versatility in curriculum design inherently accommodates learner diversity and can be adapted for use in a variety of social, situational, and ethical contexts. Methodologically, it “creates a transparency and articulation of perspectives, thoughts, and wonderings, purposefully creating self-reflexive reconstruction.”^30^

**Table 1:** The principles of duoethnography

Tenet	Definition
1. Currere	An autobiographical process of examining curriculum as a lived embodied experience.
2. Polyvocal and Dialogic	Dialogue between participants is multi-voiced and conversational.
3. Disrupts Meta-narratives	Challenges and potentially disrupts metanarratives by questioning held beliefs.
4. Differences	Participants juxtapose their different social, cultural and political orientation to articulate different experiences of a common phenomenon.
5. Transformative	Meaning moves beyond context and temporality towards that of transformation.
6. Self-Reflexivity	Knowledge is legitimized through conversations examined as contextualized beliefs or hypothetical/truthful fictions.
7. Accessibility	Text reads as storied conversations rather than academic dissertations.
8. Ethical Stances	Centered on a foundation of care and trust that respects participants as both learners and teachers in the process of curriculum exploration.

(*Note: Adapted from Norris, Sawyer & Lund, 2012*).

The first principle (or tenet) of duoethnography is its notion of *currere*, a term that describes the autobiographical process of examining curriculum as a lived embodied experience.^32^ In currere, the “duoethnographer’s [participant’s] life embodies a living, breathing curriculum where life stories become the site of the research.”^29^ Next, duoethnography is *dialogic and polyvocal* in nature.^28-30^ This multiplicity of perspective invites extrospection that *disrupts metanarratives* (the third principle) through a negotiated consideration of participants’ *differences* (the fourth principle). Participants learn to question their held beliefs by juxtaposing their social, cultural, and political lenses into the conversation “to make explicit how different people can experience the same phenomenon differently.”^29^ Its fifth and sixth principles, *transformation* and *self-reflexivity*, lead to its final two tenets, *accessibility* and its *ethical stances*.^29^ As part of this self-reflective process, “change and transformation are integral to the duoethnography”.^29^ Duoethnographers share temporal memories of past events told in the present, which transforms in meaning through self-inquiry. This knowledge is legitimized in the “depth of researcher involvement with and accompanying praxis related to her or his study,” and allows for conversations to be examined as contextualized beliefs or hypothetical/’truthful fictions’.”^29^ Since duoethnographies are discussions about self experiences *with* others, its ethical stance is centered on a foundation of care and trust that respects participants as both learners and teachers in the transformative and self-reflective process of curriculum exploration.

### Duoethnography in resident professionalism and self-care education

Medical residency was once viewed as a time to “survive” rather than a time to “thrive,” but now most residency training programs in Canada and abroad, recognize that the residency years are a critical time for self-reflection and development of affective learning skills (e.g., values, ethics, integrity, humility, professionalism, self-efficacy, etc.).^19^ As such, formal opportunities for reflective practice are being incorporated into medical and resident programs. For example, the McMaster Internal Medicine (IM) Residency Program designates protected time in the form of weekly academic half-days when residents are released from clinical responsibilities.^33^ During these academic half-days, residents participate in case-based learning (in small-group tutorials of approximately six to eight), for competency-based learning that extends beyond the CanMEDS role of medical expert (i.e., communicator, collaborator, manager, health advocate, scholar, and professional).^33^ In addition to these types of reflective forums, the McMaster IM Residency Program organizes resident retreats, as week-long and two-day events for first-year and senior residents.^33^ During these retreats, residents are exposed to subject learning in the areas of professionalism, communication, and collaboration, such as conflict/time management, medical bioethics, end-of-life care, patient safety, and personal health and wellness.^33^ From a practical perspective, these learning forums create the possibility to include duoethnography as a pedagogical model for resident professionalism and self-care education.

**Figure 1:**
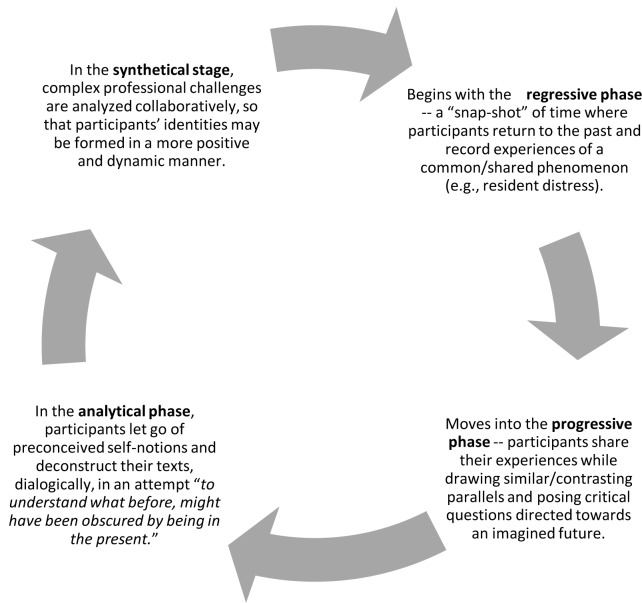
The Duoethnographic stages of professional identity currere

So, what might a duoethnographic curriculum for resident professionalism and self-care look like in action? Participants in a small group tutorial setting start in the *regressive phase*, by taking a “snap-shot” of time and recording their experiences of a shared/common phenomenon (e.g., resident distress) by “return[ing] to the past, to capture it as it was, and as it hovers over the present.”^29^ These past experiences should be recorded using “natural, informal, everyday conversational structures [that] serve as thresholds to past experiences by providing rich details to which the Other[s] can connect.” For example, participants may write a brief narrative (paragraph or two) or merge, within the text, social artifacts such as photos, poetry, song lyrics, or excerpts of the written text itself, to create different entry points, for dialogic analysis that “promote researchers’ [participants’] development of higher forms of consciousness.” [See Figure 1]^29,31^

Next, moving into the *progressive phase*, participants share their experiences while drawing similar/contrasting parallels and posing critical questions directed towards an imagined future.

As participants record their thoughts looking forward, “at what is not yet the case, what is not yet present. … [they begin to] discern where [their] intellectual interests are going, the relation between these evolving interests and [their] private [lives].”^32^ In the *analytical phase*, participants must learn to *let go* of preconceived self-notions, so that they may begin to deconstruct their texts, dialogically, in an attempt “to understand what before, might have been obscured by being in the present.”^32^ In this space, *feelings of ugliness* (shame, anger, or fear--”fear in confronting the truth of one’s past, fear of seeing the past reflected in the present, and fear of an uncertain future,”^31^) may surface, as participants may write about challenging professional situations that have contributed to personal experiences of distress. For many, this struggle may be related to their inability to connect theory with self-practice. For example, although residents and physicians are trained to recognize and treat the signs and symptoms of psychological distress in their patients, they are often at a loss when it comes to applying these practice skills to themselves.^4,13,15^ Different than other forms of reflective practice that focuses on *introspection*, an examination of one’s own conscious beliefs and emotions, duoethnography invites *extrospection*. Extrospection is an examination of the self through observations that are external to one’s mind. As such, in the *synthetical stage*, as complex professional challenges are systematically revealed, highlighted and analyzed collaboratively, a participant’s professional identity may be formed in a more positive and dynamic manner. This reconstructed identity is not formed individually, but rather, it is formed dialogically and requires careful consideration and response to the ethical dimensions of professionalism in and of itself (i.e., trust, integrity, honesty, altruism, humility, and respect).^20,31^ The opportunity to express and critically reflect upon one’s feelings in a supportive and caring environment has been shown to be a protective factor towards resident distress and a positive predictor of academic performance and professional competency development.^4^ As dialogic conversations continue to evolve in depth and understanding, residents can expand their subjectivity. Faculty and mentors can facilitate these conversations by sharing their own stories of personal and professional growth while embedding topics that may be challenging to debate in traditional instruction forums.

### Conclusion

Medicine is an intensely personal profession. Learning and teaching the profession through collaborative inquiry creates opportunities for self-reflexive and transformative educational experiences that get carried forward into residents’ careers. The context and subtext of those experiences are enacted on many different levels, as residents’ identities shift from student-learners to practitioner-leaders. Successful professional identity development enables residents to become “wholly engaged physicians”^14^ who are cognisant of and connected to the care needs of both their patients and themselves. Managing the complex and sometimes perilous dynamics of this professional growth is challenging for many, especially due to the unique pressures that residency training has on personal health and well-being. For this reason, duoethnography, a dialogic and collaborative form of curriculum inquiry, has been presented as a pedagogical model for resident professionalism and self-care education. Merits of duoethnography centers on its: 1) capability to foster self-reflexive and transformative learning; 2) versatility to accommodate learner diversity; and 3) adaptability for use in different social, situational, and ethical contexts.
